# Association of *β-Catenin*, *APC*, *SMAD3/4*, *Tp53*, and *Cyclin D1* Genes in Colorectal Cancer: A Systematic Review and Meta-Analysis

**DOI:** 10.1155/2022/5338956

**Published:** 2022-08-17

**Authors:** Hongfeng Yan, Fuquan Jiang, Jianwu Yang

**Affiliations:** Department of General Surgery, PLA Strategic Support Force Characteristic Medical Center, Beijing 100101, China

## Abstract

**Objectives:**

Accumulating evidence indicates that the expression and/or variants of several genes play an essential role in the progress of colorectal cancer (CRC). The current study is a meta-analysis undertaken to estimate the prognosis and survival associated with *CTNNB1/β-catenin*, *APC*, *Wnt*, *SMAD3/4*, *TP53*, and *Cyclin D1* genes among CRC patients.

**Methods:**

The authors searched PubMed, EMBASE, and Science Direct for relevant reports published between 2000 and 2020 and analyzed them to determine any relationship between the (immunohistochemically/sequencing-detected) gene expression and variants of the selected genes and the survival of CRC patients.

**Results:**

The analysis included 34,074 patients from 64 studies. To evaluate association, hazard ratios (HRs) were estimated for overall survival (OS) or disease-free survival (DFS), with a 95% confidence interval (CIs). Pooled results showed that *β-catenin* overexpression, *APC* mutation, *SMAD-3* or 4 loss of expression, *TP53* mutations, and *Cyclin D1* expression were associated with shorter OS. *β-Catenin* overexpression (HR: 0.137 (95% CI: 0.131–0.406)), loss of expression of *SMAD3* or 4 (HR: 0.449 (95% CI: 0.146–0.753)), the mutations of *TP53* (HR: 0.179 (95% CI: 0.126–0.485)), and *Cyclin D1* expression (HR: 0.485 (95% CI: 0.772–0.198)) also presented risk for shorter DFS.

**Conclusions:**

The present meta-analysis indicates that overexpression or underexpression and variants of *CTNNB1/β-catenin*, *APC*, *SMAD3/*4, *TP53*, and *Cyclin D1* genes potentially acted as unfavorable biomarkers for the prognosis of CRC. The *Wnt* gene was not associated with prognosis.

## 1. Introduction

Globally, cancer is the second leading cause of death after heart disease, and it is a prominent health issue. More specifically, colorectal cancer (CRC) is the third leading cause of death among men and women [1]. Unlike many other types of cancer, the survival rate for CRC has not changed a great deal. Recent studies showed that the prognostication of CRC depends upon the clinicopathological factors and the stages of tumor characteristics and reported the association with survival times and clinical outcomes [2–4]. Several susceptibility studies on the association of a genetic variant and CRC have been reported [5]. The solid tumors of CRC have served as genetic and biological paradigms and instigated to conduct studies on early detection [6], prevention [7], risk stratification [8], and treatments [9]. However, a greater understanding and identification of genetic biomarkers involving molecular and genetic pathways with improved sensitivity and specificity could improve screening for and expedite the diagnosis of CRC, yielding better outcomes. Currently, the prediction of outcomes in CRC relies heavily on traditional cancer characterization methods, including clinicopathological characteristics, such as staging, tumor size, invasion, tumor sidedness, and metastasis. It contributes to CRC's high mortality rate and tendency for poor prognosis with disappointing survival rates [10].

The uses of molecular prognostic biomarkers to forecast the progression of the condition and likely survival have interested scholars for some time [11]. However, CRC is a very diverse disease, and it is associated with complex interactions between genetic biomarkers and environmental risk factors. In addition, transduction pathways, namely transforming growth factor *β*-suppressor of mothers against decapentaplegic (*TGFβ-SMADs*), wingless/integrated (*Wnt*), and tumor suppressor protein (p53), play an essential role in the initiation and development of CRC [4]. The tumor protein p53 gene (*Tp53*) located at chromosome 17p13 consists of 90% of missense mutations. Furthermore, studies have reported that genetic variations, particularly at codon 72 Pro/Arg gene polymorphism of the *Tp53* gene, could affect the prognosis and treatment of CRC [12]. The *Wnt* signaling pathway is of particular interest because of its vital function in embryogenesis and tissue homeostasis. Many studies have identified the excessive activation of *Wnt* signaling as playing a major role in CRC [13]. A genome-scale analysis has recognized that 90% of patients with CRC carried genetic variations in the *Wnt* signaling pathway, particularly the loss-of-functional variations of adenomatous polyposis coli (*APC*) and variations that activate the mutations of *β-catenin* [14].

The membranous expression of *β-catenin* applies a restrictive impact on the movements of tumor cells and their growth. The increases in cell motility, growth, and transformation promote tumorigenesis because of the loss of *β-catenin* expression on the cell surface [12]. Pre-existing intracellular *β-catenin* can cause abnormality in *Wnt*/*β-catenin*-TCF signaling, leading to the progression of CRC. The hyperactivation of Wnt/*β*-catenin signaling enhances the invasive and metastatic possibility of CRC cells, while the knockdown of *β-catenin* in CRC cells reduces cell proliferation and further invasion [15]. Studies have reported the detection of nuclear *β-catenin* expression using immunohistochemical methods, and they have reported an association with a high burden of tumor and poor CRC survival [15].

Somatic mutations at the *APC* gene are found in approximately 75% of CRC cases. Several studies have suggested worse outcomes for CRC patients with wild-type *APC* (*APC*-WT) in comparison to mutant-type *APC* (*APC*-MT) [16]. However, the prognostic implication of this genomic alteration is not well-defined, especially in metastatic CRCs. SMAD4/DPC4 is a tumor suppressor gene that regulates cell growth and a common intracellular mediator that could alter the *TGFβ* signaling to promote tumor progression. Studies have reported an association of SMAD4 genetic variation with tumor invasion, metastasis, and prognosis in various cancers [17].

In light of inconsistent results in the literature, the authors perceived a need for a meta-analysis that would explore the prognostic value of selected genes in CRC. The objectives were to estimate the pooled risk (hazard ratio, HR) identified (between the years 2000 and 2020) for each of these genes for overall survival (OS) and disease-free survival (DFS) in CRC patients. Thus, this meta-analysis comprehensively explores the prognostic role of selected genes in the *β-catenin* and related pathway implicated in the development and progression of CRC.

## 2. Methods

### 2.1. Publication Search and Inclusion Criteria

The authors searched the databases of PubMed, EMBASE and Science Direct for relevant published articles. Search terms included medical phrases related to *SMAD 3*, *SMAD 4*, *β-catenin*, Catenin beta 1(*CTNNB1*), *APC*, *Wnt*, *Cyclin D1*, *Tp53*, or p53 genes and their variants/polymorphisms, in combination with words related to CRC (tumor, neoplasms, carcinoma, CRC, colon cancer, or rectal cancer). In addition, terms related to prognosis (outcome or survival) were used to retrieve eligible studies from 2000 through to the end of 2020. Furthermore, the references in the selected published articles were searched to identify potentially relevant studies.

Eligible studies were selected based on the following criteria: (a) pathologically confirmed (i.e., via tissue samples) patients with CRC, (b) immunohistochemical/sequencing detection methods for the selected genes and OS, DFS, cancer-specific survival (CSS), or recurrence-free survival (RFS), (c) English language, and (d) full-text articles. Editorial letters, reviews, case reports, studies with duplicated/repeated data, and studies lacking essential information and animal studies were excluded.

### 2.2. Data Extraction

In accordance with the meta-analysis of observational studies in epidemiology (MOOSE) guidelines [18] and in compliance with PRISMA guidelines, the data were evaluated and extracted by two independent researchers, who entered them all onto the data extraction form. For data extraction, the details recorded were as follows: the first author, publication year, country, total number of cases, type of cancer, stages, reported genes, gene detection method, cut-off values used, hazard ratios (HRs) with their 95% confidence intervals (CIs), and *P* values. For inconsistencies, a consensus was reached on each item among the authors. The Newcastle–Ottawa scale (NOS) was used to evaluate the quality of the eligible studies.

### 2.3. Statistical Analysis

The meta-analysis was executed based on HRs calculated by the log‐rank test for OS and RFS differences with different gene expression levels. Calculations were based on HRs from the original publications, including 95% CI, and subsequent back-calculation to log (HR) and standard error (SE) for overall estimates. Wherever available, HRs based on a multivariate analysis were used. Log (HR) and SE were entered in statistical software NCSS (NCSS, LLC, Kaysville, UT, https://www.ncss.com/), and meta-analyses were validated in the software Comprehensive Meta‐Analysis (CMA; Biostat, Inc., Englewood, NJ, https://www.meta-analysis.com/). The heterogeneity of pooled results was analyzed using Cochran's *Q* test and the Higgins I-squared statistic. The absence of heterogeneity is based on the *Q* test revealed P heterogeneity>0.1 and *I*^2^ < 50%. To estimate the summary HRs/ORs, a fixed-effects model (the Mantel–Haenszel method) was used [19]. Elsewhere, the arandom-effects model (the DerSimonian and Laird method) [20] was used. To examine the publication bias, Begg's funnel plot and Egger's linear regression test were used, and *P* < 0.05 was considered statistically significant (i.e., an asymmetrical distribution). All of the results were presented with HRs, upper and lower limits, and *P* values and were illustrated in forest plots for the individual studies with the weighted and pooled effects.

## 3. Results

### 3.1. Study Characteristics

**Figure 1:**
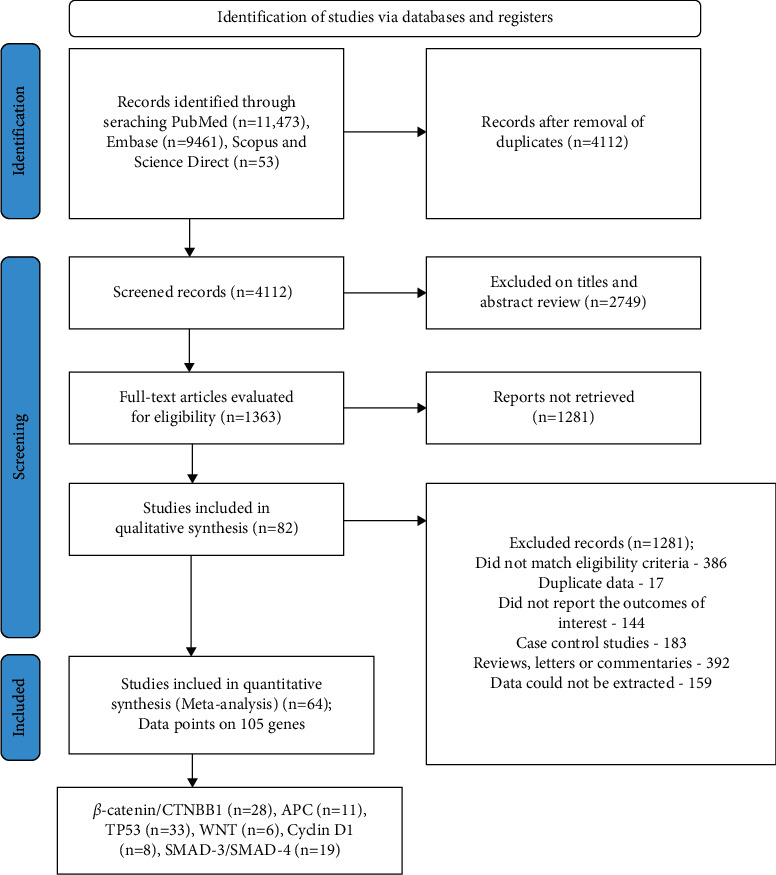
PRISMA flow chart of the selected studies.

Figure 1 shows the comprehensive process used to select articles in this study, which was based on PRISMA guidelines. After the removal of duplicates, the database search yielded 4,112 articles. Based on the inclusion criteria and after screening the titles, abstracts, figures, and key data, 82 articles were finalized for literature studies [21–40], [41–60], [61–80], [81–102]. However, only 64 articles [21–31, 33–36, 38–40, 42–56, 59–61, 64, 66, 68–70, 72, 73, 75, 76, 78, 81–86, 88, 90, 91, 93, 95, 97–102] were retrieved for meta-analysis with 105 data points of the selected genes. Of these, four studies had evaluated the prognostic value for RFS [47, 81, 88, 101]. Six studies included cancer-specific survival [26, 46, 48, 65, 98, 103], whereas three reported progression-free survival (PFS) [32, 76, 84]. All others reported either OS and/or DFS. Since the number of studies for the first three indicators was small, the data for CSS, PFS, and RFS were combined with DFS. Thus, 64 studies involving 34,074 patients evaluating OS and DFS were analyzed in the current meta-analysis.

### 3.2. Review of Eligible Studies

The 82 studies identified as having presented data on baseline genes and prognosis in CRC are listed in Table 1 [21–40], [41–60], [61–80], [81–102]. Most of these studies were from the USA (*n* = 18), followed by China (*n* = 11), Korea (*n* = 7), Sweden (*n* = 6), Japan and Greece (*n* = 5), Australia and Austria (*n* = 4), Norway (*n* = 3), Taiwan, Egypt, Germany, Hungary, Italy, Netherlands and Turkey (*n* = 2), and one each from Brazil, France, Hungary, Iran, Poland, Romania, Scotland, Spain, and Switzerland. Two studies were multicentric [45, 52]. The number of patients ranged from 39 [93] to 3,583 [45]. Patients were diagnosed with CRC (*n* = 59), rectal cancer (*n* = 7), and colon cancer (*n* = 12). The data presented in these studies were on the *Wnt* gene (*n* = 6), *β-catenin* or *CTNNB1* (*n* = 28), *Tp53* or p53 (*n* = 33), *APC* (*n* = 11), *SMAD* (19), and *Cyclin-D1* (*n* = 8), with some studies including data on multiple genes (Figure 1). The extraction procedure in all studies was carried out using IHC on tissue samples. The tumors were most commonly graded according to TNM or Dukes' classification, which is 14.9% [71] to 69.4% [59] of the right-sided tumors.

### 3.3. Quality of Eligible Studies

The Newcastle–Ottawa Scale (NOS) was used to examine the methodological quality of the included studies. As previously described, a score of 9 implied the highest quality, while a score of ≥5 was considered to be high quality. Seventy-two studies included in our meta-analysis were of high quality, i.e., they had scores of 5 or more after quality assessment.

### 3.4. Prognostic Value of Gene Expression and Mutations in Colorectal Cancer

**Figure 2:**
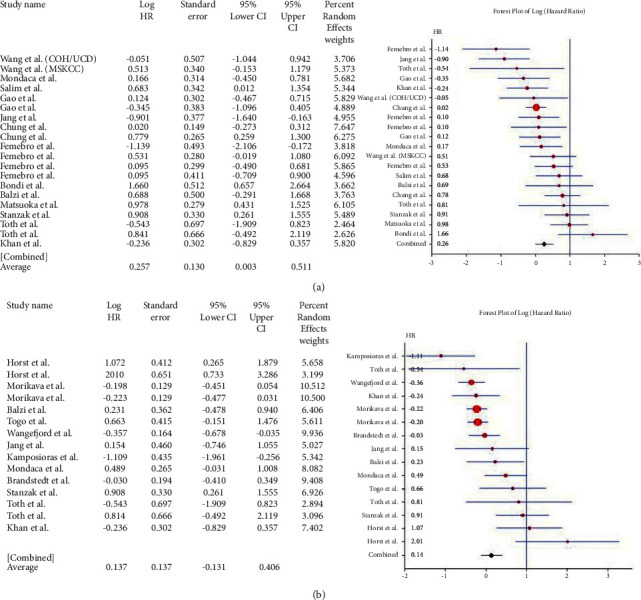
Forest plot of *β*-catenin gene and overall survival in CRC (a). Forest plot of *β*-catenin gene and disease-free survival in CRC (b).

Sixty-five studies, with 105 data points on genes where HR data was available, were included in the meta-analysis. These are shown in Table 2. Twenty-eight enrolled studies provided the HRs, and 95% CI directly or indirectly reported the correlation between *β-catenin* overexpression and OS. The pooled HR of *β-catenin* overexpression in the nucleus, cytoplasm, or membranous with OS was 0.257 (95% CI: 0.003–0.511; *Q* = 53.978; *P* = 0.000) (Figure 2(a)), however, heterogeneity existed. The association of *β-catenin* overexpression with shorter DFS was analyzed. The pooled HR was 0.137 (95% CI: 0.131–0.406; *Q* = 48.832; *P* = 0.000) (Figure 2(b)). The above results suggested that *β-catenin* overexpression in the nucleus, membrane, or cytoplasm was associated with lower OS and DFS.

**Figure 3:**
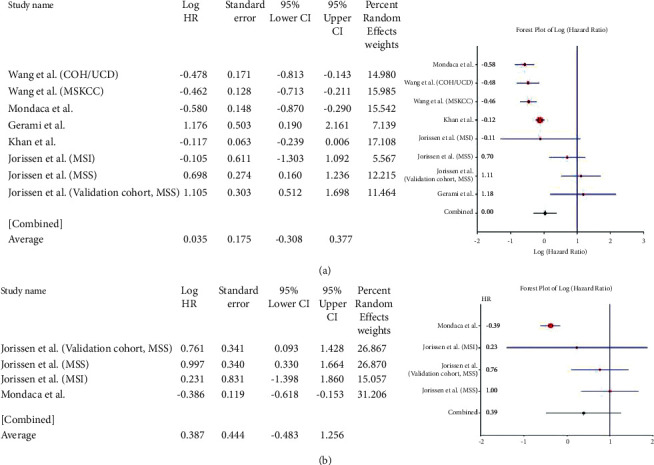
Forest plot of *APC* gene and overall survival in CRC (a). Forest plot of *APC* gene and disease-free survival in CRC (b).

**Figure 4:**
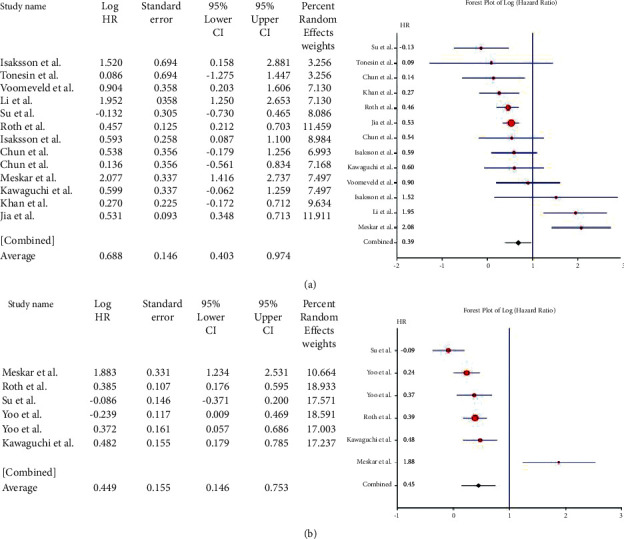
Forest plot of *SMAD3/4* gene and overall survival in CRC (a). Forest plot of *SMAD3/4* gene and disease-free survival in CRC (b).

For the *APC* gene, the pooled HR for OS based on 8 studies was 0.035 (95% CI: 0.308–0.377; *Q* = 51.76; *P*=0.000) (Figure 3(a)). This value suggested the association of the mutant variant with a lower OS compared with the wild type but not for DFS, where pooled HR = 0.387 (95% CI: 0.483–1.256; *Q* = 22.624; *P*=0.000) (Figure 3(b)). For the *SMAD3/4* genes, 13 studies were included. The pooled HR was 0.688 (95% CI: 0.403–0.974; *Q* = 47.689; *P*=0.000) (Figure 4(a)). Their pooled HR for DFS was 0.449 (95% CI: 0.146–0.753; *Q* = 32.012; *P*=0.000) (Figure 4(b)). These results implied a worse prognosis of CRC in the event of the loss of expression of *SMAD-3* or *SMAD*-4.

**Figure 5:**
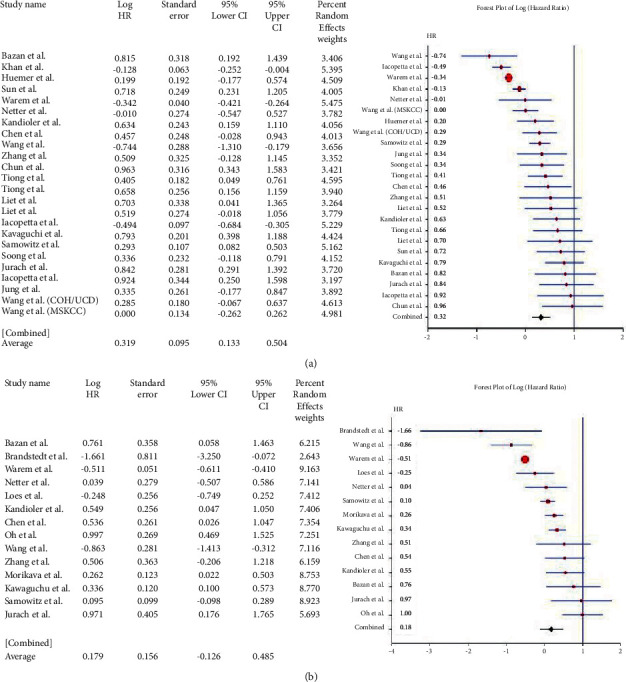
Forest plot of *TP53* gene and overall survival in CRC (a). Forest plot of *TP53* gene and disease-free survival in CRC (b).

**Figure 6:**
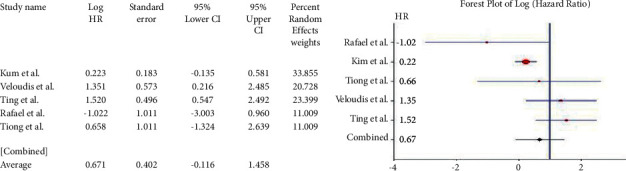
Forest plot of *WNT* gene and overall survival in CRC.

**Figure 7:**
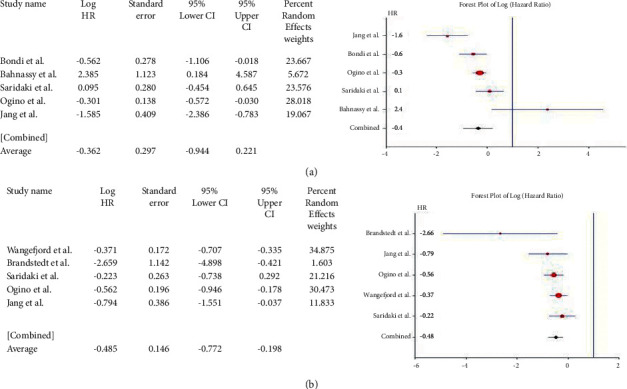
Forest plot of *Cyclin D1* gene and overall survival in CRC (a). Forest plot of *cyclin D1* gene and disease-free survival in CRC (b).

Studies reporting the mutations of the *Tp53* gene (*n* = 24) had a pooled HR of 0.319 (95% CI: 0.133–0.504; *Q* = 201.339; *P*=0.000) (Figure 5(a)) for OS and 0.179 (95% CI: 0.126–0.485; *Q* = 143.796; *P*=0.000) (Figure 5(b)) for DFS (*n* = 14). The results were widely heterogenous but implied significantly poor prognosis overall, as well as DFS, in CRC cases. Five studies showed a pooled HR of 0.671 (95% CI: 0.116–1.458; *Q* = 10.746; *P*=0.030) (Figure 6) for the *Wnt* gene with OS, thereby showing no association of *Wnt* gene expression/mutation with survival in CRC. Since only one study [14] reported the hazard ratio for DFS, meta-analysis was not performed for the *Wnt* gene with shorter DFS. Five studies on *Cyclin D1* were included in the meta-analysis. The pooled HR for OS was 0.362 (95% CI: 0.944–0.221; *Q* = 5.421; *P*=0.253) (Figure 7(a)) and that for DFS was 0.485 (95% CI: 0.772–0.198; *Q* = 5.810; *P*=0.214) (Figure 7(b)). High *Cyclin D1*, therefore, produced a worse prognosis in CRC, both in terms of OS and DFS.

### 3.5. Publication Bias

**Figure 8:**
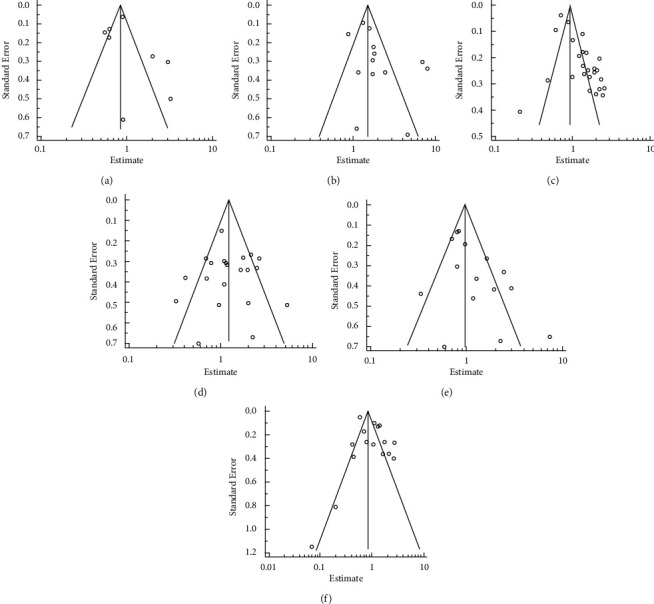
The funnel plot of studies included for *APC* gene and OS in CRC (a). The funnel plot of studies included for *SMAD* gene and OS in CRC (b). The funnel plot of studies included for *β*-catenin gene and OS in CRC (c). The funnel plot of studies included for *β*-catenin gene and DFS in CRC (d). The funnel plot of studies included for *TP53* gene and OS in CRC (e). The funnel plot of studies included for *TP53* gene and DFS in CRC (f).

We assessed the publication bias for *APC*, *SMAD*, *β-catenin*, and *Tp53* gene studies by constructing funnel plots (Figure 8(a)–8(f)) as more than ten studies were included in the meta-analysis. Egger's test indicated that publication bias existed for the evaluation of the impact of *β*-catenin, *APC*, and *Tp53* with OS, however, Begg's test showed no significant publication bias (*β-catenin* and OS: I^2^ = 65.83%, tau (*τ*) = 0.047 (*P*=0.76), *β-catenin* and DFS: *I*^2^ = 71.33%, *τ* = 0.21 (*P*=0.25), *TP53* and OS: *I*^2^ = 88.82%, *τ* = 0.153 (*P*=0.28), *TP53* and DFS: *I*^2^ = 89.12%, *τ* = 0.25 (*P*=0.13), *APC* and OS: *I*^2^ = 86.48%; *τ* = 0.28 (*P*=0.32), *SMAD* and OS: *I*^2^ = 83.17%, and *τ* = 0.23 (*P*=0.27)). It is notable that with Egger's test, there is insufficient power of testing when the number of selected studies is below 20. It was, therefore, not attempted for the remaining genes.

## 4. Discussion

Colorectal carcinogenesis is a complex multistage process that involves multiple genetic variations. The aberrant activation of the *Wnt*/*β-catenin* pathway has been identified as being involved in the progression of CRC [104] and early colorectal tumorigenesis [103]. In several studies, the *β-catenin* accumulation in the nucleus or cytoplasm was identified as a marker for poor prognosis. The variations of the *APC* or *CTNNB1* genes are the main causes of the accumulation of nuclear *β-catenin* [105]. In contrast, *β-catenin* expression in the nucleus was associated with noninvasive tumors and more favorable outcomes [106] but remains controversial.

The current meta-analysis has explored the cumulative prognostic significance of the different subcellular localizations of *β-catenin* expression among CRC subjects. The results indicated that the nuclear expression or decreased expression of *β-catenin* in the membrane was associated with lower OS, which is consistent with the published articles. Pooled data from a study [107] found that the reduced expression of *β-catenin* in the membrane to be significantly associated with poor survival among CRC patients, thus the majority of the selected studies are from nuclear *β-catenin* overexpression.

*Wnt*2 is an oncogene with the potential to activate canonical *Wnt* signaling during CRC tumorigenesis [21, 22]. The role of *Wnt*5 in the progression of CRC is quite complex and appears to be inconsistent in findings. Several studies [21–25] proved that *Wnt*5a was silenced in most CRC cell-lines because of recurrent methylation in the promoter region. *Wnt*5a acts as a tumor suppressor by interfering with the canonical *β-catenin* signaling. However, it activates the noncanonical signaling pathways [100]. In this study, there was no significant association of *Wnt* (2 and 5) to OS or DFS found among CRC patients, and it is well in accordance with the contradictory studies reported [23–25].

In our meta-analysis pertaining to *SMAD* genes, we found that the loss of *SMAD* 3 or *SMAD*4 staining was strongly associated with a worse prognosis for OS and DFS (including CSS/RFS). Several other individual reports are in alignment with our findings [87, 92, 93]. These studies reported *SMAD*-4 to have a stronger association compared with *SMAD*-3 or other *SMAD* genes.

Most studies have shown the predictive value of *Tp53* for overall survival in CRC to be poor. Dong et al. [108] reported 53% of *Tp53* gene variation as the susceptibility for the development of CRC. Another study reported that, in mouse models, a high rate of spontaneous tumors was noted because of p53-deficiency [109]. Moreover, the deletion of p53 and the *Tp53* gene variation led to tumor progression and tumor cell death.

A meta-analysis of Asian patients indicates that an association between *Tp53* Arg72Pro polymorphism CC genotype might contribute to an increased risk of CRC [110]. The current meta-analysis included diverse populations, and the results pertaining to the association of *Tp53* with shorter overall and DFS in CRCs may, therefore, be considered more generalizable.

In an independent study of 331 patients, the prognostic value of *APC* was evaluated, and the findings were validated on a public database of stage IV colon cancer from Memorial Sloan Kettering Cancer Center (MSKCC) [75]. The study found that *APC*-WT was present in 26% of metastatic CRC patients, and it was more prevalent in patients of younger age and those with right-sided tumors. *APC*-WT tumors have been shown to be associated with other *Wnt*-activating alterations, including *CTNNB1, FBXW7, RNF43, ARID1A,* and *SOX9*. *APC*-WT patients in a study were found to have a worse overall survival (OS) than *APC*-MT pts (HR = 1.809, 95% CI: 1.260–2.596) [75]. Overall, in most studies, *APC*-WT is associated with poor OS. Additionally, *APC*-WT tumors were associated with other activating alterations of the *Wnt* pathway, including RNF43 and *CTNNB1*.

*Cyclin D1* overexpression has been reported to occur in 40–70% of colorectal tumors [111]. Despite the well-established role of *Cyclin D1* in cell cycle progression, previous data on *Cyclin D1* and clinical outcomes in CRC have been conflicting. *Cyclin D1* overexpression has also been significantly related to poor OS in Asian and non-Asian CRC patients [112]. Two mechanisms have been implicated, namely nuclear expression and cytoplasmic expression, wherein most studies found an association of the nuclear expression of *Cyclin D1* with OS and DFS. Moreover, *Cyclin D1* also has been shown as a poor prognosis marker when co-expressed with other genes, notably p53 [113]. These results are consistent with the present meta-analysis's findings that shortened overall survival and DFS are associated with *Cyclin D1* among CRC patients.

We acknowledge that this study has several limitations. Firstly, the element of bias cannot be ruled out because of the inclusion of retrospective studies. Secondly, all of the selected studies measured gene expression by immunohistochemistry and sequencing methods. Moreover, the cut-offs used in various studies differed between and across the genes studied. However, there was no subgroup analysis performed to investigate the potential effect of the technique on the combined results. Thirdly, some heterogeneity has been found because of location and the types of cancer. To eliminate variations across studies, a random-effects model was performed accordingly. Limited databases were used for article search, and only freely available full-text articles in the English language were used, which might affect the persuasive power of the pooled estimate, although to a limited extent. In addition, publication bias existed because only studies generating positive results or significant outcomes were suitable for publication. Future research might helpfully contribute further relevant analyses and well-designed extensive prospective studies, since they will address the limitations of the current meta-analysis.

## 5. Conclusion

The present meta-analysis has found that the genes associated with worst OS in CRC were *β-catenin* (cytoplasmic, membranous, and nuclear overexpression), *APC* (mutant type), *Tp53* (mutated), *SMAD-3* and *SMAD*-4 (loss of expression), and *Cyclin D1* (high). The gene associated with shorter DFS in CRC patients was *APC* (mutant type). In contrast, *Wnt* (2 and 5) genes were not associated with prognosis in CRC in this meta-analysis.

## Figures and Tables

**Table 1:** Characteristics of included studies.

No.	Author	Year	Region	Sample size	Male %	Sample type	Tumor type	Clinical stage of tumor	Tumor side (right %)	Gene	Method of gene expression	Elevated levels/abnormality	Cut-off value	Outcome	NOS rating
1	Rafael et al. [21]	2014	Spain	345	53.3	Tissue	CRC	Duke A-D	NA	Wnt	SSCP	Mutations	NA	*β*-Catenin mutation not associated with OS	5
2	Yoshida et al. [22]	2015	Japan	201	59.7	Tissue	CRC	Stage 1,2,3	NA	Wnt	IHC	High, low	>50%	Nuclear *β*-catenin associated with poor OS and DFS	6
3	Ting et al. [23]	2013	Taiwan	282	52.4	Tissue	CRC	AJCC	NA	Wnt	Genomic DNA sequencing, tagger algorithm	Polymorphism	NA	*Wnt* polymorphism associated with high risk in OS	6
4	Veloudis et al. [24]	2017	Greece	57	NA	Tissue	Colon and rectal adenocarcinoma	TNM 1–4	33.3	Wnt	IHC	Negative, weak, intermediate, strong	Median	Nuclear *β*-catenin associated with poor OS	5
5	Kim et al. [25]	2018	Korea	194	65.5	Tissue	CRC	NA	22.2	Wnt 5A	Genomic DNA extraction	Methylated/nonmethylated	NA	Methylation observed in 32%, not associated with OS	7
6	Wangefjord et al. [26]	2011	Austria	527	47.6	Tissue	CRC	TNM 1–4	NA	Cyclin D1	IHC	weak, moderate, strong	>0–>75%	High Cyclin D1 expression associated with poor survival in men	7
7	Bazan et al. [27]	2005	Italy	160	47.5	Tissue	CRC	Duke A-D	NA	TP53	PCR-SSCP	Mutation	NA	Associated with poor OS	6
8	Khan et al. [28]	2018	USA	1825	56.7	Tissue	CRC	NA	37.2	TP53	Genomic sequencing	Mutation	5–10%	Associated with poor OS	5
										CTNNB1					
										SMAD-4					
										APC					
9	Brandstedt et al. [29]	2014	Sweden	304	0	Tissue	CRC	TNM 1–4	NA	p53	IHC staining and gene sequencing	Positive/negative	p53: >50%; *β*-catenin: 0–2; Cyclin D1: 0->75%	Associated with poor OS	6
10	Huemer et al. [30]	2018	Austria	161	39.7	Tissue	CRC	Grade 1–3	24	TP53	Genomic DNA sequencing	Mutation	NA	TP53 mutation not associated with shorter OS compared with TP53 wild type tumor. TP53 mutation not associated with shorter OS in right-sided tumors	5
11	Sun et al. [31]	2014	China	197	64.4	Tissue	CRC	TNM 0–4	NA	TP53	IHC	High/low	150	Associated with poor OS	5
12	Theodoropoulos et al. [32]	2008	Greece	165	67.8	Tissue	Colorectal adeno cancer	TNM stage 1–4	NA	TP53	Nuclear immunostaining of positive cells	Overexpression	>10%	p53+: 63.5% tumors. Advanced *T* stage associated with p53 expression	4
13	Warren et al. [33]	2013	USA	607	55.5	Tissue	Colon cancer	Stage 3	NA	TP53	Direct sequencing and hybridization	Mutation	NA	TP53 mutations- 45%	4
14	Netter et al. [34]	2014	France	68	75	Tissue	Colon ca., metastatic	NA	67.6	TP53	FASAY and sanger sequencing	NA	10–15%	Associated with poor OS	5
15	Kandioler et al. [35]	2015	Austria	389	51.1	Tissue	Colon cancer	Stage 3	NA	TP53	Sanger sequencing	Mutations	<75%	Associated with poor OS	4
16	Chen et al. [36]	2013	China	203	42.3	Tissue	CRC	AJCC	NA	TP53	IHC	Negative, positive	>10%	Associated with poor OS	5
17	Russo et al. [37]	2014	USA	222	26.12	Tissue	CRC	Stage 1–4	NA	TP53, APC	Clinical tumor genotyping	Mutations	NA	TP53 mutations: 21% APC mutations: 8%	5
18	Oh et al. [38]	2019	Korea	621	59.9	Tissue	CRC	AJCC 2 and 3	NA	TP53	IHC and next generation sequencing	Weak, moderate, strong	0%	Weak expression associated with poor OS	6
19	Wang et al. [39]	2017	China	124	50.8	Tissue	CRC	TNM 1–4	NA	TP53	IHC	Expression	>10%	P53 positive: 58.8%	7
20	Zhang et al. [40]	2014	China	185	42.7	Tissue	CRC	AJCC 1–4	40	TP53	IHC	Negative/positive	<10% cells with +ve nuclei: Negative; >10% cells with +ve nuclei: Positive	Associated with poor OS	7
21	Godai et al. [41]	2009	Japan	211	57.8	Tissue	CRC	Duke stage A-D	NA	TP53	Genomic DNA Sequencing	Mutations	NA	TP53 mutations: 70%	6
22	Chun et al. [42]	2019	USA	401	55.6	Tissue	CRC	AJCC	24.6	TP53 APC SMAD-4	Next gen sequencing	Low or high risk (EAp53 score)	NA	TP53 mutations: 65.6% APC mutations: 47.4% SMAD-4 mutations: 11.4%	8
23	Tiong et al. [43]	2014	China and taiwan	NA	NA	Tissue	CRC	NA	NA	TP53, CTNNB1, Wnt 5A	IHC	Overexpression	NA	Associated with poor survival	4
24	Li et al. [44]	2018	China	315	57.1	Tissue	CRC	TNM	NA	TP53	Next gen mutational analysis	Mutation	NA	Double mutated P53 with PIK3CA associated with poor survival	6
25	Iacopetta et al. [45]	2006	Multinational	3583	52.3	Tissue	CRC	Dukes stage A-D	NA	TP53	PCR	Mutation	NA	TP53 mutation associated with distal colon cancer	6
26	Morikawa et al. [46]	2012	USA	1060	39	Tissue	Colon and rectal cancer	Stages 1–4	NA	TP53	IHC	Moderate and strong	NA	Associated with poor OS	8
27	Kawaguchi et al. [47]	2019	USA	490	58.3	Tissue	CRC	AJCC Cat. T	NA	TP53SMAD-4	Nextgen sequencing	Expression	>10%	Associated with poor OS	7
28	Samowitz et al. [48]	2002	USA	1464	50.2	Tissue	Colon cancer	AJCC	NA	TP53	NA	NA	NA	Associated with poor survival	7
29	Soong et al. [49]	2000	Australia	995	NA	Tissue	CRC	Duke stage B&C	34	TP53	NA	Mutation	NA	39% mutations	5
30	Jurach et al. [50]	2006	Brazil	83	56.6	Tissue	Rectal	Astler Coller B&C	NA	TP53	IHC	Mutation	>20%	Associated with poor OS	5
31	Loes et al. [51]	2016	Norway	151	60.2	Tissue	CRC	NA	NA	TP53	Sanger sequencing	Mutations	NA	TP53 mutations- 60.4%	4
32	Iacopetta et al. [52]	2006	Multinational	3583	52.3	Tissue	CRC	Dukes stage A-D	NA	TP53	PCR	Mutation	NA	TP53 mutation associated with distal colon cancer	6
33	Salim et al. [53]	2013	Sweden	85	NA	Tissue	Colon cancer	NA	NA	*β*-Catenin	IHC	Less expression	<50%	Associated with poor OS	4
34	Kamposioras et al. [54]	2013	Greece	106	61.3	Tissue	CRC	NA	40% (CRC)	*β*-Catenin	IHC	Overexpression	Moderate	Associated with poor OS	7
35	Gao et al. [55]	2014	China	181	58	Tissue	CRC	TNM stages 1–4	NA	*β*-Catenin	IHC	Overexpression	>50%	Associated with poor OS	6
36	Jang et al. [56]	2012	Korea	218	61.4	Tissue	Colon cancer	NA	23.3	*β*-Catenin, Cyclin D1	IHC	Overexpression	>30%	Associated with poor survival	5
37	Lee et al. [57]	2013	Korea	305	61.9	Tissue	CRC	AJCC stages 1–4	NA	*β*-Catenin	IHC	Overexpression	>30%	Associated with poor OS	6
38	Wong et al. [58]	2003	China	60	65	Tissue	CRC	NA	NA	*β*-Catenin	IHC	Overexpression	>300	Associated with poor survival	4
39	Chung et al. [59]	2001	USA	543	NA	Tissue	CRC	NA	NA	*β*-Catenin	IHC	Overexpression	Moderate	Associated with poor survival	4
40	Fernebro et al. [60]	2004	Sweden	257	67.3	Tissue	Rectal cancer	NA	NA	*β*-Catenin, p53	IHC	Abnormal expression	Weak	Associated with poor survival	5
41	Bondi et al. [61]	2004	Norway	162	45.6	Tissue	colon cancer	NA	NA	*β*-Catenin	IHC	overexpression	>1%	Associated with poor survival	4
42	Kim et al. [62]	2005	Korea	124	NA	Tissue	CRC	Duke A-D	NA	*β*-Catenin	IHC	Abnormal expression	>5%	Associated with poor survival	6
43	Filiz et al. [63]	2010	Turkey	138	60.1	Tissue	CRC	NA	NA	*β*-Catenin	IHC	Expression levels	Weak	Associated with poor survival	5
44	Jung et al. [64]	2013	Korea	349	59.5	Tissue	CRC	NA	21.7	*β*-Catenin, p53	IHC	Overexpression	>0%	Associated with poor survival	7
45	Wangefjord et al. [65]	2013	Sweden	527	47.4	Tissue	CRC	TNM stages 1–4	NA	*β*-Catenin	IHC	Overexpression	Moderate	Associated with poor survival	5
46	Balzi et al. [66]	2015	Italy	321	53.2	Tissue	CRC	NA	NA	*β*-Catenin	IHC	Overexpression	Moderate	Associated with poor survival	5
47	Youssef et al. [67]	2015	Egypt	72	48.1	Tissue	CRC	TNM stages 1–4 and dukes A-C	69.4	*β*-Catenin	IHC	Overexpression	>10%	Associated with poor survival	6
48	Togo et al. [68]	2008	USA	183	62.8	Tissue	CRC	TNM stages 1–4	33.3	*β*-Catenin, p53	IHC	Overexpression	Moderate/strong expression	Associated with poor survival	5
49	Matsuoka et al. [69]	2011	Japan	156	63.4	Tissue	CRC	TNM stages 1–4	NA	*β*-Catenin	IHC	Overexpression	>20%	Associated with poor survival	7
50	Morikawa et al. [70]	2011	USA	955	39.9	Tissue	CRC	NA	NA	*β*-Catenin	IHC	Overexpression	Moderate/strong expression	Associated with poor survival	8
51	Ozguven et al. [71]	2011	Turkey	60	33.3	Tissue	CRC	NA	NA	*β*-Catenin	IHC	overexpression	>0%	Associated with poor survival	5
52	Stanczak et al. [72]	2011	Poland	66	66.66	Tissue	CRC	NA	NA	*β*-Catenin	IHC	Overexpression	>10%	Associated with poor survival	6
53	Toth et al. [73]	2012	Hungary	79	50.6	Tissue	CRC	NA	NA	*β*-Catenin	IHC	Overexpression	>10%	Associated with poor survival	7
54	Sun et al. [74]	2011	China	67	64.2	Tissue	Colon cancer	NA	NA	*β*-Catenin	IHC	Decreased expression	>10%	Downregulation associated with increased expression of E-Cadherin	8
55	Wang et al. [75]	2020	USA	341 (COH)	56.3	Tissue	COAD	NA	30.7	APC TP53 CTNNB1	DNA sequencing	Mutations	NA	APC mutations- 74.8%	8
				934 (MSKCC)	52.9	Tissue		NA	26.1	APC TP53 CTNNB1	DNA sequencing	Mutations	NA	APC mutations- 74.8%	
56	Mondaca et al. [76]	2020	USA	471		Tissue	CRC	NA	32%	APC CTNNB1	Tumor genomic profiling	Expression	NA	APC associated with poor survival	7
57	Schell et al. [77]	2016	USA	407	NA	Tissue	CRC	NA	41	APC	TGS	Mutation	NA	Associated with poor survival	4
58	Gerami et al. [78]	2020	Iran	57	77.2	Frozen tissue	CRC	TNM stage 1 to 4	36.8	APC	DNA sequencing	AG vs. AA genotype	NA	AG genotype associated with poor survival	5
59	Conlin et al. [79]	2005	Scotland	107	60.7	Tissue	CRC	Duke stage A-D	14.9	APC p53	Genomic DNA extraction and sequencing	Mutations	NA	APC mutations: 56%; p53 mutations: 61%; not associated	4
60	Wang et al. [80]	2020	USA	331	NA	Microsatellite stable, tissue	CRC	4	NA	APC	Next-gen genomic analysis	APC –WT or APC-MT	NA	APC-WT associated with poor survival	7
61	Jorissen et al. [81]	2015	Australia	746	55.4	CRC MSI (unstable) and MSS (stable); validation cohort, tissue	CRC	Stage 1 to 4	42.2	APC TP53	DNA sequencing	APC-WT or APC-MT	NA	TP53: 55.4%; APC-WT associated with poor survival	6
62	Voorneveld et al. [82]	2012	Netherlands	209	NA	Tissue	CRC	NA	NA	SMAD-4	IHC	Expression	NA	Associated with poor survival	5
63	Li et al. [83]	2011	China	147	NA	Tissue	CRC	NA	NA	SMAD-4	IHC	Expression	NA	Associated with poor survival	5
64	Yoo et al. [84]	2019	Korea	1370	NA	Tissue	CRC	NA	NA	SMAD-4	NA	SMAD-4 high vs. low	NA	Associated with poor survival	5
65	Su et al. [85]	2016	China	251	57.37	Tissue	CRC	Stages 1–4	NA	SMAD-4	NA	SMAD-4 positive	NA	No association	5
66	Isaksson et al. [86]	2006	Sweden	86	42	Tissue	CRC	Duke A-C	35	SMAD-4	IHC	Negative – 3+	NA	Associated with poor OS	6
67	Fleming et al. [87]	2013	Australia	744	55.6	Sporadic CRCs, tissue	CRC	AJCC stages 1–4	42.07	SMAD-4	IHC	Stroma high, stroma low	NA	Associated with poor survival	4
68	Roth et al. [88]	2012	Switzerland	1404	NA	Tissue	CRC	Stage 2 (18%) and 3 (23%)	NA	SMAD-4	IHC detection	Loss of expression	NA	Associated with poor survival	6
69	Lampropoulos et al. [89]	2012	Greece	195	NA	Tissue	CRC	Stage 1 to 4	NA	SMAD-4	NA	NA	NA	Associated with poor survival	4
70	Isaksson et al. [90]	2011	Sweden	441	NA	Tissue	CRC	Stage 1 to 4	NA	SMAD-4	IHC	Loss, moderate, high	0–5%	Loss of SMAD—24%; associated with poor OS	5
71	Jia et al. [91]	2017	US	209	51.7	Tissue	CRC	Stages 1–4	NA	SMAD-4	Genomic DNA sequencing	High, low	NA	High cytoplasm and low nuclear SMAD-4 not associated with OS	7
72	Oyanagi et al. [92]	2019	Japan	201	117	Tissue	CRC	TNM 1–4	56	SMAD-4	IHC	Weak, strong	>95%	SMAD-4 alterations: 28%, associated with poor OS and RFS	6
73	Ionescu et al. [93]	2014	Romania	39	66.6	Tissue	CRC	Duke A-D	25.6	SMAD-3	q-RT-PCR	Overexpression, under-expression	NA	No association with OS	6
74	Fukushima et al. [94]	2003	Japan	100	NA	Sporadic CRC and normal tissue	Sporadic CRC	NA	NA	SMAD3/SMAD4	PCR-SSCP	Abnormal	NA	SMAD-3: no abnormality; SMAD-4: abnormal 5 cases	4
75	Chun et al. [95]	2014	Korea	201	65.7	Tissue	Rectal cancer	3	NA	SMAD4	PCR	Nuclear or cytoplasmic SMAD-4	NA	No association	6
76	Bacman et al. [96]	2007	Germany	310	61	Tissue	Colon cancer	Stage 2 (57.4%) and 3 (42.6%)	NA	SMAD3/SMAD4	PCR	SMAD-3 and SMAD-4 in tumor high or low	NA	SMAD-3 and SMAD-4 in tumor, effects on TGF*β* R2 pathway downregulation	5
77	Meskar et al. [97]	2009	Netherlands	135	54.4	Tissue	CRC	Stage 1 (17.8%), 2 (77.8%) and 3 (4.4%)	53.3	SMAD4	NA	Stroma high vs. stroma low	NA	Stroma high SMAD-4 associated with poor prognosis	7
78	Horst et al. [98]	2009	Germany	142	50	Tissue	CRC	UICC stage 2A	NA	*β*-Catenin	IHC staining	Nuclear *β*-catenin	NA	Associated with poor survival	6
79	Bondi et al. [99]	2005	Norway	219	47.9	Tissue	Colon cancer	Duke A-D	NA	Cyclin D1	Real time q-PCR and IHC	Low, high	Grade +2	Cyclin not associated with survival.	6
80	Bahnassy et al. [100]	2004	Egypt	60	60.0	Tissue	CRC	TNM 1–4	NA	Cyclin D1	DNA extraction and gene amplification, IHC	amplification	>75%	Associated with poor survival	7
81	Saridaki et al. [101]	2010	Greece	144	56.94	Tissue	CRC	Stages 1–4	NA	Cyclin D1	DNA extraction and IHC	Weak, strong	≥50% with weak and ≥20% with strong staining	Overexpression is not associated with poor outcomes	6
82	Ogino et al. [102]	2009	USA	602	43	Tissue	Colon cancer	AJCC stages 1–4	NA	Cyclin D1	IHC	No, weak, moderate, strong	Strong staining in any fraction	Overexpression not associated with poor survival	8

NA: not applicable; CRC: colon rectal cancer; COAD: colon adenocarcinoma; IHC: immunohistochemical; OS: overall survival.

**Table 2:** Hazard ratios of studies included in meta-analysis.

No.	Author	Year	Gene	Outcome	HR	95% CI	
						Lower	Upper
1	Wang et al. (COH/UCD) [75]	2020	APC	OS	0.62	0.44	0.86
	Wang et al. (MSKCC) [75]		APC	OS	0.63	0.49	0.81
	Wang et al. (COH/UCD) [75]		CTNNB1	OS	0.95	0.35	2.55
	Wang et al. (MSKCC) [75]		CTNNB1	OS	1.67	0.86	3.26
	Wang et al. (COH/UCD) [75]		TP53	OS	1.33	0.93	1.88
	Wang et al. (MSKCC) [75]		TP53	OS	1.00	0.77	1.30
2	Mondaca et al. [76]	2020	APC	Progression-free survival	0.68	0.54	0.86
				OS	0.56	0.42	0.75
			CTNNB1	Progression-free survival	1.63	0.97	2.74
				OS	1.18	0.64	2.19
3	Gerami et al. [78]	2020	APC	OS	3.24	1.21	8.68
4	Jorissen et al. (MSI) [81]	2015	APC	OS	0.90	0.27	2.96
				RFS	1.26	0.25	6.50
	Jorissen et al. (MSS) [81]	2015	APC	OS	2.01	1.17	3.43
				RFS	2.71	1.39	5.28
	Jorissen et al. (Validation cohort, MSS) [81]	2015	APC	OS	3.02	1.67	5.47
				RFS	2.14	1.10	4.18
5	Voorneveld et al. [82]	2012	SMAD-4	OS	2.47	1.02	4.15
6	Li et al. [83]	2011	SMAD-4	OS	7.04	3.88	12.82
7	Yoo et al. [84]	2019	SMAD-4	Progression-free survival	1.27	1.01	1.60
				Cancer-free survival	1.45	1.06	1.99
8	Su et al. [85]	2016	SMAD-4	DFS	0.92	0.69	1.222
				OS	0.87	0.64	1.187
9	Roth et al. [88]	2012	SMAD-4	OS	1.58	1.23	2.01
				RFS	1.47	1.19	1.81
10	Isaksson et al. [90]	2011	SMAD-4	OS	1.81	1.09	3.00
11	Chun et al. [95]	2014	SMAD-4 (nuclear)	OS	1.71	0.83	3.511
			SMAD-4 (cytoplasmic)	OS	1.15	0.57	2.30
12	Meskar et al. [97]	2009	SMAD4	OS	7.98	4.12	15.44
				DFS	6.57	3.43	12.56
13	Salim et al. [53]	2013	*β* catenin (membrane *β-*catenin absent + nuclear GSK3 *β*)	OS	1.98	1.01	3.89
14	Kamposioras et al. [54]	2013	*β*-Catenin (membrane)	DFS	0.33	0.14	0.77
15	Gao et al. [55]	2014	*β*-Catenin (membrane)	OS	1.13	0.62	2.05
			*β*-Catenin (nucleus)	OS	0.71	0.38	1.70
16	Jang et al. [56]	2012	*β*-Catenin	OS	0.41	0.19	0.85
				DFS	1.16	0.47	2.85
	Jang et al. [56]	2012	Cyclin D1	OS	0.205	0.09	0.46
				DFS	0.45	0.21	0.96
17	Chung et al. [59]	2001	*β*-Catenin, nuclear	OS	1.02	0.73	1.31
			*β*-Catenin, phosphonuclear	OS	2.18	1.30	3.68
18	Fernebro et al. [60]	2004	*β*-Catenin (cytoplasm)	OS	0.32	0.12	0.83
			*β*-Catenin (membrane)	OS	1.7	1.00	3.0
	Fernebro et al. [60]	2004	*β*-Catenin (nucleus)	OS	1.1	0.62	2.0
			p53	OS	1.1	0.50	2.5
19	Bondi et al. [61]	2004	*β*-Catenin (nuclear, combined with C-Myc)	OS	5.26	1.93	14.36
20	Jung et al. [64]	2013	*β*-Catenin	OS	0.68	0.39	1.19
			p53	OS	1.39	0.82	2.28
21	Wangefjord et al. [65]	2013	*β*-Catenin	Cancer-specific survival	0.70	0.51	0.97
22	Balzi et al. [66]	2015	*β*-Catenin (nucleus)	OS	1.99	0.75	5.32
				DFS	1.26	0.62	2.56
23	Togo et al. [68]	2008	*β*-Catenin	DFS	1.94	0.86	4.38
			p53	DFS	1.70	0.83	3.48
24	Matsuoka et al. [69]	2011	*β*-Catenin	OS	2.66	1.54	4.60
25	Morikawa et al. [70]	2011	*β*-Catenin (cytoplasm)	Cancer-specific mortality	0.82	0.64	1.06
			*β*-Catenin (nucleus)	Cancer-specific mortality	0.80	0.62	1.03
26	Stanzak et al. [72]	2011	*β*-Catenin	OS	2.48	1.30	4.74
27	Toth et al. [73]	2012	*β*-Catenin (membrane)	OS	0.58	0.14	2.28
			*β*-Catenin (nucleus)	OS	2.25	0.61	8.32
28	Horst et al. [98]	2009	*β*-Catenin	DFS	2.92	1.30	6.53
				Cancer-specific survival	7.46	2.08	26.72
29	Bazan et al. [27]	2005	TP53	OS	2.26	1.21	4.21
				DFS	2.14	1.06	4.32
30	Khan et al. [28]	2018	TP53	OS	0.88	0.78	1.00
			CTNNB1	OS	0.79	0.44	1.44
			SMAD-4	OS	1.31	1.09	1.57
			APC	OS	0.89	0.79	1.01
31	Brandstedt et al. [29]	2014	p53	CRC Risk	0.19	0.04	0.96
			*β*-Catenin	CRC risk	0.97	0.66	1.41
			Cyclin D1	CRC risk	0.07	0.01	0.88
32	Huemer et al. [30]	2018	TP53	OS	1.22	0.84	1.78
33	Sun et al. [31]	2014	TP53	OS	2.05	1.26	3.34
34	Warren et al. [33]	2013	TP53	OS	0.71	0.65	0.76
				DFS	0.60	0.54	0.66
35	Netter et al. [34]	2014	TP53	OS	0.99	0.53	1.55
				Progression-free survival	1.04	0.60	1.79
36	Loes et al. [51]	2016	TP53	Disease-specific survival	0.78	0.47	1.28
37	Kandioler et al. [35]	2015	TP53	OS	1.88	1.17	3.04
				CFS	1.73	1.04	2.86
38	Chen et al. [36]	2013	TP53	OS	1.58	0.97	2.56
				DFS	1.71	1.03	2.86
39	Oh et al. [38]	2019	TP53	5-year survival	2.71	1.60	4.60
40	Wang et al. [39]	2017	TP53	OS	0.47	0.27	0.83
				DFS	0.42	0.24	0.73
41	Zhang et al. [40]	2014	TP53	OS	1.66	0.88	3.14
				DFS	1.65	0.81	3.38
42	Chun et al. [42]	2019	TP53	OS	2.62	1.41	4.87
43	Tiong et al. [43]	2014	TP53 (and CTNNB1)	OS	1.50	1.05	2.14
			Wnt 5A	OS	1.93	1.17	3.19
44	Li et al. [44]	2018	TP53 (double mutation with PIK3CA)	OS	2.02	1.04	3.91
			TP53	OS	1.68	0.98	2.87
45	Morikawa et al. [46]	2012	TP53	Cancer-specific survival	1.30	1.02	1.65
46	Kawaguchi et al. [47]	2019	TP53	OS	2.21	1.49	3.28
				RFS	1.40	1.11	1.78
			SMAD-4	OS	1.82	1.17	2.83
				RFS	1.62	1.20	2.20
47	Samowitz et al. [48]	2002	TP53	OS	1.34	1.07	1.63
				Cancer-specific survival	1.10	0.91	1.34
48	Soong et al. [49]	2000	TP53	OS	1.40	0.89	2.21
49	Jurach et al. [50]	2006	TP53	OS	2.32	1.34	4.03
				Recurrence	2.64	1.19	5.83
50	Iacopetta et al. [45]	2006	TP53	OS	2.52	1.28	4.93
51	Iacopetta et al. [52]	2006	TP53	OS	0.61	0.50	0.73
52	Wangefjord et al. [26]	2011	Cyclin D1	Cancer-specific survival	0.69	0.49	0.96
53	Isaksson et al. [86]	2006	SMAD-4	OS	4.57	1.17	17.8
54	Tonescu et al. [93]	2014	SMAD-3	OS	1.09	0.30	3.99
55	Jia et al. [91]	2017	SMAD-4 (nuclear)	OS	1.70	0.96	3.00
			SMAD-4 (cytoplasm)	OS	1.39	0.76	2.56
56	Kim et al. [25]	2018	Wnt	OS	1.25	0.87	1.78
57	Veloudis et al. [24]	2017	Wnt/*β*-catenin	OS	3.86	1.24	11.9
58	Ting et al. [23]	2013	Wnt	OS	4.57	1.73	12.1
59	Yoshida et al. [22]	2015	Wnt	DFS	1.50	0.80	2.8
			*β*-Catenin	DFS	2.10	1.10	3.9
				OS	1.90	1.00	3.4
60	Rafael et al. [21]	2014	Wnt	OS	0.36	0.05	2.63
61	Bondi et al. [99]	2005	Cyclin D1	OS	0.57	0.33	0.98
62	Bahnassy et al. [100]	2004	Cyclin D1	OS	10.86	1.05	86.2
63	Saridaki et al. [101]	2010	Cyclin D1	OS	1.1	0.6	1.8
				RFS	0.8	0.5	1.4
64	Ogino et al. [102]	2009	Cyclin D1	OS	0.74	0.57	0.98
				CSS	0.57	0.39	0.84

The table represents 105 data points on genes where HR data were available. OS: overall survival, RFS: relapse-free survival, CFS: cancer-free survival, DFS: disease-free survival, PFS: progression-free survival, CRC risk: colorectal cancer risk.

## Data Availability

The data extraction sheets used to support the findings of this study are available from the corresponding author upon request.

## Abbreviations

APC: Adenomatous polyposis coli
ARID1A: AT-rich interaction domain 1A
CIs: Confidence intervals
CRC: Colorectal cancer
CSS: Cancer-specific survival
DFS: Disease-free survival
CTNNB1: Catenin beta 1
FBXW7: F-box and WD repeat domain containing 7
HRs: Hazard ratios
OS: Overall survival
p53: Tumor suppressor protein
PFS: Progression-free survival
RNF43: Ring finger protein 43
RFS: Recurrence-free survival
SMAD: Suppressor of mothers against decapentaplegic
SOX9: SRY-box transcription factor 9
Tp53: Tumor protein p53 gene
TGF*β*: Transforming growth factor *β*
Wnt: Wingless/integrated.

## References

[1] Rawla P., Sunkara T., Barsouk A. (2019). Epidemiology of colorectal cancer: incidence, mortality, survival, and risk factors. *Przeglad Gastroenterolgiczny*.

[2] Mohd Y., Balasubramanian B., Meyyazhagan A. (2021). Extricating the association between the prognostic factors of Colorectal Cancer. *Journal of Gastrointestinal Cancer*.

[3] Li J., Ma X., Chakravarti D., Shalapour S., DePinho R. A. (2021). Genetic and biological hallmarks of colorectal cancer. *Genes & Development*.

[4] Luo X. J., Zhao Q., Liu J. (2021). Novel genetic and epigenetic biomarkers of prognostic and predictive significance in stage ii/iii colorectal cancer. *Molecular Therapy*.

[5] Labadie J. D., Savas S., Harrison T. A. (2022). Genome-wide association study identifies tumor anatomical site-specific risk variants for colorectal cancer survival. *Scientific Reports*.

[6] El Kadmiri N. (2021). Advances in early detection of colorectal cancer: a focus on non-invasive biomarkers. *Current Drug Targets*.

[7] Patel S. G., Karlitz J. J., Yen T., Lieu C. H., Boland C. R. (2022). The rising tide of early-onset colorectal cancer: a comprehensive review of epidemiology, clinical features, biology, risk factors, prevention, and early detection. *The Lancet Gastroenterology & Hepatology*.

[8] Archambault A. N., Jeon J., Lin Y. (2022). Risk stratification for early-onset colorectal cancer using a combination of genetic and environmental risk scores: an international multi-center study. *Journal of the National Cancer Institute*.

[9] Biller L. H., Schrag D. (2021). Diagnosis and treatment of metastatic colorectal cancer: a review. *Journal of the American Medical Association*.

[10] Hassan M. R. A., Suan M. A. M., Soelar S. A., Mohammed N. S., Ismail I., Ahmad F. (2016). Survival analysis and prognostic factors for colorectal cancer patients in Malaysia. *Asian Pacific Journal of Cancer Prevention: Asian Pacific Journal of Cancer Prevention*.

[11] Koncina E., Haan S., Rauh S., Letellier E. (2020). Prognostic and predictive molecular biomarkers for colorectal cancer: updates and challenges. *Cancers*.

[12] Ahmad R., Singh J. K., Wunnava A., Al‑Obeed O., Abdulla M., Srivastava S. (2021). Emerging trends in colorectal cancer: dysregulated signaling pathways (review). *International Journal of Molecular Medicine*.

[13] Steinhart Z., Angers S. (2018). Wnt signaling in development and tissue homeostasis. *Development*.

[14] Schatoff E. M., Leach B. I., Dow L. E. (2017). Wnt signaling and colorectal cancer. *Current Colorectal Cancer Reports*.

[15] Cheng X., Xu X., Chen D., Zhao F., Wang W. (2019). Therapeutic potential of targeting the Wnt/*β*-catenin signaling pathway in colorectal cancer. *Biomedicine & Pharmacotherapy*.

[16] Bruun J., Kolberg M., Nesland J. M., Svindland A., Nesbakken A., Lothe R. A. (2014). Prognostic significance of *β*-catenin, E-cadherin, and SOX9 in colorectal cancer: results from a large population-representative series. *Frontiers in Oncology*.

[17] Liu S., Chen S., Zeng J. (2018). TGF *β* signaling: a complex role in tumorigenesis (review). *Molecular Medicine Reports*.

[18] Stroup D. F., Berlin Ja J. A., Mortan S. C. (2000). Meta-analysis of observational studies in epidemiology: a proposal for reporting. Meta-analysis of observational studies in epidemiology (MOOSE) group. *Journal of the American Medical Association*.

[19] Mantel N., Haenszel W. (1959). Statistical aspects of the analysis of data from retrospective studies of disease. *Journal of the National Cancer Institute*.

[20] DerSimonian R., Laird N. (1986). Meta-analysis in clinical trials. *Controlled Clinical Trials*.

[21] Rafael S., Veganzones S., Vidaurreta M., de la Orden V., Maestro M. L. (2014). Effect of *β*-catenin alterations in the prognosis of patients with sporadic colorectal cancer. *Journal of Cancer Research and Therapeutics*.

[22] Yoshida N., Kinugasa T., Ohshima K. (2015). Analysis of Wnt and beta-catenin expression in advanced colorectal cancer. *Anticancer Research*.

[23] Ting W. C., Chen L. M., Pao J. B. (2013). Common genetic variants in wnt signaling pathway genes as potential prognostic biomarkers for colorectal cancer. *PLoS One*.

[24] Veloudis G., Pappas A., Gourgiotis S. (2017). Assessing the clinical utility of wnt pathway markers in colorectal cancer. *Journal of BUON*.

[25] Kim S. H., Park K. H., Shin S. J. (2018). CpG island methylator phenotype and methylation of wnt pathway genes together predict survival in patients with colorectal cancer. *Yonsei Medical Journal*.

[26] Wangefjord S., Manjer J., Gaber A., Nodin B., Eberhard J., Jirstrom K. (2011). Cyclin D1 expression in colorectal cancer is a favorable prognostic factor in men but not in women in a prospective, population-based cohort study. *Biology of Sex Differences*.

[27] Bazan V., Agnese V., Corsale S. (2005). Specific TP53 and/or Ki-ras mutations as independent predictors of clinical outcome in sporadic colorectal adenocarcinomas: results of a 5-year gruppo oncologico dell’Italia meridionale (GOIM) prospective study. *Annals of Oncology*.

[28] Khan M., Loree J. M., Advani S. M. (2018). Prognostic implications of mucinous differentiation in metastatic colorectal carcinoma can be explained by distinct molecular and clinicopathologic characteristics. *Clinical Colorectal Cancer*.

[29] Brandstedt J., Wangefjord S., Nodin B., Eberhard J., Jirstrom K., Manjer J. (2014). Associations of hormone replacement therapy and oral contraceptives with risk of colorectal cancer defined by clinicopathological factors, beta-catenin alterations, expression of cyclin D1, p53, and microsatellite-instability. *BMC Cancer*.

[30] Huemer F., Thaler J., Piringer G. (2018). Sidedness and TP53 mutations impact OS in anti-EGFR but not anti-VEGF treated mCRC-an analysis of the KRAS registry of the AGMT (arbeitsgemeinschaft medikamentöse tumortherapie). *BMC Cancer*.

[31] Sun R., Wang X., Zhu H. (2014). Prognostic value of LAMP3 and TP53 overexpression in benign and malignant gastrointestinal tissues. *Oncotarget*.

[32] Theodoropoulos G. E., Karafoka E., Papailiou J. G. (2009). p53 and egfr expression in colorectal cancer: a reappraisal of “old” tissue markers in patients with long follow-up. *Anticancer Research*.

[33] Warren R. S., Atreya C. E., Niedzwiecki D. (2013). Association of TP53 mutational status and gender with survival after adjuvant treatment for stage III colon cancer: results of CALGB 89803. *Clinical Cancer Research*.

[34] Netter J., Lehmann-Che J., Lambert J. (2015). Functional TP53 mutations have no impact on response to cytotoxic agents in metastatic colon cancer. *Bulletin du Cancer*.

[35] Kandioler D., Mittlböck M., Kappel S. (2015). TP53 mutational status and prediction of benefit from adjuvant 5-fluorouracil in stage III colon cancer patients. *EBioMedicine*.

[36] Chen J., Tang H., Wu Z. (2013). Overexpression of RBBP6, alone or combined with mutant TP53, is predictive of poor prognosis in colon cancer. *PLoS One*.

[37] Russo A. L., Borger D. R., Szymonifka J. (2014). Mutational analysis and clinical correlation of metastatic colorectal cancer. *Cancer*.

[38] Oh H. J., Bae J. M., Wen X. (2019). p53 expression status is associated with cancer-specific survival in stage III and high-risk stage II colorectal cancer patients treated with oxaliplatin-based adjuvant chemotherapy. *British Journal of Cancer*.

[39] Wang P., Liang J., Wang Z., Hou H., Shi L., Zhou Z. (2017). The prognostic value of p53 positive in colorectal cancer: a retrospective cohort study. *Tumor Biology*.

[40] Zhang M., Cui F., Lu S. (2014). Increased expression of prothymosin-*α*, independently or combined with TP53, correlates with poor prognosis in colorectal cancer. *International Journal of Clinical and Experimental Pathology*.

[41] Godai T. I., Suda T., Sugano N. (2009). Identification of colorectal cancer patients with tumors carrying the TP53 mutation on the codon 72 proline allele that benefited most from 5-fluorouracil (5-FU) based postoperative chemotherapy. *BMC Cancer*.

[42] Chun Y. S., Passot G., Yamashita S. (2019). Deleterious effect of RAS and evolutionary high-risk TP53 double mutation in colorectal liver metastases. *Annals Of Surgery*.

[43] Tiong K. L., Chang K. C., Yeh K. T. (2014). CSNK1E/CTNNB1 are synthetic lethal to TP53 in colorectal cancer and are markers for prognosis. *Neoplasia*.

[44] Li A. J., Li H. G., Tang E. J. (2018). PIK3CA and TP53 mutations predict overall survival of stage II/III colorectal cancer patients. *World Journal of Gastroenterology*.

[45] Iacopetta B., Russo A., Bazan V. (2006). Functional categories of TP53 mutation in colorectal cancer: results of an International collaborative Study. *Annals of Oncology*.

[46] Morikawa T., Kuchiba A., Liao X. (2012). Tumor TP53 expression status, body mass index and prognosis in colorectal cancer. *International Journal of Cancer*.

[47] Kawaguchi Y., Kopetz S., Newhook T. E. (2019). Mutation status of RAS, TP53, and SMAD4 is superior to mutation status of RAS alone for predicting prognosis after resection of colorectal liver metastases. *Clinical Cancer Research*.

[48] Samowitz W. S., Curtin K., Ma K. N. (2002). Prognostic significance of p53 mutations in colon cancer at the population level. *International Journal of Cancer*.

[49] Soong R., Powell B., Elsaleh H. (2000). Prognostic significance of TP53 gene mutation in 995 cases of colorectal carcinoma. *European Journal of Cancer*.

[50] Jurach M. T., Meurer L., Moreira L. F. (2006). Expression of the p53 protein and clinical and pathologic correlation in adenocarcinoma of the rectum. *Arquivos de Gastroenterologia*.

[51] Løes I. M., Immervoll H., Sorbye H. (2016). Impact of KRAS, BRAF, PIK3CA, TP53 status and intraindividual mutation heterogeneity on outcome after liver resection for colorectal cancer metastases. *International Journal of Cancer*.

[52] Iacopetta B., Russo A., Bazan V. (2005). TP53-CRC Collaborative Study Group. The TP53 colorectal cancer international collaborative study on the prognostic and predictive significance of p53 mutation: influence of tumor site, type of mutation, and adjuvant treatment. *Journal Of Clinical Oncology*.

[53] Salim T., Sjolander A., Sand-Dejmek J. (2013). Nuclear expression of glycogen synthase kinase-3*β* and lack of membranous *β*-catenin is correlated with poor survival in colon cancer. *International Journal of Cancer*.

[54] Kamposioras K., Konstantara A., Kotoula V. (2013). The prognostic significance of WNT pathway in surgically-treated colorectal cancer: beta-catenin expression predicts for disease-free survival. *Anticancer Research*.

[55] Gao Z. H., Lu C., Wang M. X., Han Y., Guo L. J. (2014). Differential beta-catenin expression levels are associated with morphological features and prognosis of colorectal cancer. *Oncology Letters*.

[56] Jang K. Y., Kim Y. N., Bae J. S. (2012). Expression of Cyclin D1 is associated with beta-catenin expression and correlates with good prognosis in colorectal adenocarcinoma. *Translational Oncology*.

[57] Lee S. J., Choi S. Y., Kim W. J. (2013). Combined aberrant expression of E-cadherin and S100A4, but not beta-catenin is associated with disease-free survival and overall survival in colorectal cancer patients. *Diagnostic Pathology*.

[58] Wong S. C. C., Lo E. S., Chan A. K., Lee K. C., Hsiao W. L. (2003). Nuclear beta catenin as a potential prognostic and diagnostic marker in patients with colorectal cancer from Hong Kong. *Molecular Pathology*.

[59] Chung G. G., Provost E., Kielhorn E. P., Charette L. A., Smith B. L., Rimm D. L. (2001). Tissue microarray analysis of beta-catenin in colorectal cancer shows nuclear phospho-beta-catenin is associated with a better prognosis. *Clinical Cancer Research: An Official Journal of the American Association for Cancer Research*.

[60] Fernebro E., Bendahl P. O., Dictor M., Persson A., Ferno M., Nilbert M. (2004). Immunohistochemical patterns in rectal cancer: application of tissue microarray with prognostic correlations. *International Journal of Cancer*.

[61] Bondi J., Bukholm G., Nesland J. M., Bukholm I. R. K. (2004). Expression of non-membranous beta-catenin and gamma-catenin, c-Myc and Cyclin D1 in relation to patient outcome in human colon adenocarcinomas. *Acta Pathologica, Microbiologica et Immunologica Scandinavica*.

[62] Kim C. J., Cho Y. G., Park Y. G. (2005). Pin1 overexpression in colorectal cancer and its correlation with aberrant beta-catenin expression. *World Journal of Gastroenterology*.

[63] Filiz A. I., Senol Z., Sucullu I., Kurt Y., Demirbas S., Akin M. L. (2010). The survival effect of E-cadherin and catenins in colorectal carcinomas. *Colorectal Disease*.

[64] Jung W., Hong K. D., Jung W. Y. (2013). SIRT1 expression is associated with good prognosis in colorectal cancer. *Korean Journal of Pathology*.

[65] Wangefjord S., Brandstedt J., Lindquist K. E., Nodin B., Jirstrom K., Eberhard J. (2013). Associations of beta-catenin alterations and MSI screening status with expression of key cell cycle regulating proteins and survival from colorectal cancer. *Diagnostic Pathology*.

[66] Balzi M., Ringressi M. N., Faraoni P. (2015). B-cell lymphoma 2 and beta-catenin expression in colorectal cancer and their prognostic role following surgery. *Molecular Medicine Reports*.

[67] Youssef N. S., Osman W. M. (2015). Relationship between osteopontin and beta-catenin immunohistochemical expression and prognostic parameters of colorectal carcinoma. *International Journal of Clinical and Experimental Pathology*.

[68] Togo N., Ohwada S., Sakurai S. (2008). Prognostic significance of BMP and activin membrane-bound inhibitor in colorectal cancer. *World Journal of Gastroenterology*.

[69] Matsuoka T., Mitomi H., Fukui N. (2011). Cluster analysis of claudin-1 and -4, E-cadherin, and beta-catenin expression in colorectal cancers. *Journal of Surgical Oncology*.

[70] Morikawa T., Kuchiba A., Yamauchi M. (2011). Association of CTNNB1 (beta-catenin) alterations, body mass index, and physical activity with survival in patients with colorectal cancer. *Journal of the American Medical Association*.

[71] Ozguven B. Y., Karacetin D., Kabukcuoglu F., Taskin T., Yener S. (2011). Immunohistochemical study of E-cadherin and beta-catenin expression in colorectal carcinomas. *Polish Journal of Pathology: Official Journal of the Polish Society of Pathologists*.

[72] Stanczak A., Stec R., Bodnar L. (2011). Prognostic significance of wnt-1, *β*-catenin and E-cadherin expression in advanced colorectal carcinoma. *Pathology and Oncology Research*.

[73] Toth L., Andras C., Molnar C. (2012). Investigation of beta-catenin and E-cadherin expression in Dukes B2 stage colorectal cancer with tissue microarray method. Is it a marker of metastatic potential in rectal cancer?. *Pathology and Oncology Research*.

[74] Sun L., Hu H., Peng L. (2011). P-cadherin promotes liver metastasis and is associated with poor prognosis in colon cancer. *American Journal Of Pathology*.

[75] Wang C., Ouyang C., Sandhu J. S., Kahn M., Fakih M. (2020). Wild-type APC and prognosis in metastatic colorectal cancer. *Journal of Clinical Oncology*.

[76] Mondaca S., Walch H. S., Nandakumar S. (2019). Influence of WNT and DNA damage response pathway alterations on outcomes in patients with unresectable metastatic colorectal cancer. *Journal of Clinical Oncology*.

[77] Schell M., Yang M., Teer J. (2016). A multigene mutation classification of 468 colorectal cancers reveals a prognostic role for APC. *Nature Communication*.

[78] Mir Najd Gerami S., Hossein Somi M., Vahedi L., Farassati F., Dolatkhah R. (2020). The APC gene rs41115 polymorphism is associated with survival in Iranian colorectal cancer patients. *Biomedical Research and Therapy*.

[79] Conlin A., Smith F., Carey A., Wolf C. R., Steele R. J. (2005). The prognostic significance of K-ras, p53, and APC mutations in colorectal carcinoma. *Gut*.

[80] Wang C., Ouyang C., Cho M. (2021). Wild-type APC is associated with poor survival in metastatic microsatellite stable colorectal cancer. *The Oncologist*.

[81] Jorissen R. N., Christie M., Mouradov D. (2015). Wild-type APC predicts poor prognosis in microsatellite-stable proximal colon cancer. *British Journal of Cancer*.

[82] Voorneveld P. W., Jacobs R. J., De Miranda N. F. (2013). Evaluation of the prognostic value of pSMAD immunohistochemistry in colorectal cancer. *European Journal of Cancer Prevention*.

[83] Li X., Liu B., Xiao J., Yuan Y., Ma J., Zhang Y. (2011). Roles of VEGF-C and SMAD4 in the lymphangiogenesis, lymphatic metastasis, and prognosis in colon cancer. *Journal of Gastrointestinal Surgery*.

[84] Yoo S. Y., Lee J. A., Shin Y., Cho N. Y., Bae J. M., Kang G. H. (2019). Clinicopathological characterization and prognostic implication of SMAD4 expression in colorectal carcinoma. *Journal of Pathology and Translational Medicine*.

[85] Su F., Li X., You K. (2016). Expression of VEGF-D, SMAD4, and SMAD7 and their relationship with lymphangiogenesis and prognosis in colon cancer. *Journal of Gastrointestinal Surgery*.

[86] Isaksson-Mettavainio M., Palmqvist R., Forssell J., Stenling R., Oberg A. (2006). SMAD4/DPC4 expression and prognosis in human colorectal cancer. *Anticancer Research*.

[87] Fleming N. I., Jorissen R. N., Mouradov D. (2013). SMAD2, SMAD3 and SMAD4 mutations in colorectal cancer. *Cancer Research*.

[88] Roth A. D., Delorenzi M., Tejpar S. (2012). Integrated analysis of molecular and clinical prognostic factors in stage II/III colon cancer. *JNCI Journal of the National Cancer Institute*.

[89] Lampropoulos P., Zizi-Sermpetzoglou A., Rizos S., Kostakis A., Nikiteas N., Papavassiliou A. G. (2012). Prognostic significance of transforming growth factor beta (TGF-beta) signaling axis molecules and E-cadherin in colorectal cancer. *Tumor Biology*.

[90] Isaksson-Mettavainio M., Palmqvist R., Dahlin A. M. (2012). High SMAD4 levels appear in microsatellite instability and hypermethylated colon cancers, and indicate a better prognosis. *International Journal of Cancer*.

[91] Jia X., Shanmugam C., Paluri R. K. (2017). Prognostic value of loss of heterozygosity and sub-cellular localization of SMAD4 varies with tumor stage in colorectal cancer. *Oncotarget*.

[92] Oyanagi H., Shimada Y., Nagahashi M. (2019). SMAD4 alteration associates with invasive‐front pathological markers and poor prognosis in colorectal cancer. *Histopathology*.

[93] Ionescu C., Braicu C., Chiorean R. (2014). TIMP-1 expression in human colorectal cancer is associated with SMAD3 gene expression levels: a pilot study. *Journal of Gastrointestinal and Liver Diseases*.

[94] Fukushima T., Mashiko M., Takita K. (2003). Mutational analysis of TGF-beta type II receptor, SMAD2, SMAD3, SMAD4, SMAD6 and SMAD7 genes in colorectal cancer. *Journal of Experimental & Clinical Cancer Research: Climate Research*.

[95] Chun H. K., Jung K. U., Choi Y. L. (2014). Low expression of transforming growth factor beta-1 in cancer tissue predicts a poor prognosis for patients with stage III rectal cancers. *Oncology*.

[96] Bacman D., Merkel S., Croner R., Papadopoulos T., Brueckl W., Dimmler A. (2007). TGF-beta receptor 2 downregulation in tumour-associated stroma worsens prognosis and high-grade tumours show more tumour-associated macrophages and lower TGF-beta1 expression in colon carcinoma: a retrospective study. *BMC Cancer*.

[97] Mesker W. E., Liefers G. J., Junggeburt J. M. C. (2009). Presence of a high amount of stroma and downregulation of SMAD4 predict for worse survival for stage I-II colon cancer patients. *Analytical Cellular Pathology*.

[98] Horst D., Reu S., Kriegl L., Engel J., Kirchner T., Jung A. (2009). The intratumoral distribution of nuclear beta-catenin is a prognostic marker in colon cancer. *Cancer*.

[99] Bondi J., Husdal A., Bukholm G., Nesland J. M., Arne B., Bukholm I. R. K. (2005). Expression and gene amplification of primary (A, B1, D1, D3, and E) and secondary (C and H) cyclins in colon adenocarcinomas and correlation with patient outcome. *Journal of Clinical Pathology*.

[100] Bahnassy A. A., Zekri A. R. N., El-Houssini S. (2004). Cyclin A and Cyclin D1 as significant prognostic markers in colorectal cancer patients. *BMC Gastroenterology*.

[101] Saridaki Z., Papadatos-Pastos D., Tzardi M. (2010). BRAF mutations, microsatellite instability status and cyclin D1 expression predict metastatic colorectal patients’ outcome. *British Journal of Cancer*.

[102] Ogino S., Nosho K., Irahara N. (2009). A cohort study of cyclin D1 expression and prognosis in 602 colon cancer cases. *Clinical Cancer Research*.

[103] Bienz M., Clevers H. (2000). Linking colorectal cancer to wnt signaling. *Cell*.

[104] Gough N. R. (2012). Focus issue: wnt and *β*-catenin signaling in development and disease. *Science Signaling*.

[105] Morin P. J., Sparks A. B., Korinek V. (1997). Activation of beta-catenin-Tcf signaling in colon cancer by mutations in beta-catenin or APC. *Science*.

[106] Chen Z., He X., Jia M. (2013). *β*-catenin overexpression in the nucleus predicts progress disease and unfavourable survival in colorectal cancer: a meta-analysis. *PLoS One*.

[107] Zhang S., Wang Z., Shan J. (2016). Nuclear expression and/or reduced membranous expression of *β*-catenin correlate with poor prognosis in colorectal carcinoma-a meta-analysis. *Medicine*.

[108] Dong Z., Zheng L., Liu W., Wang C. (2018). Association of mRNA expression of TP53 and the TP53 codon 72 Arg/Pro gene polymorphism with colorectal cancer risk in Asian population: a bioinformatics analysis and meta-analysis. *Cancer Management Research*.

[109] Donehower L. A., Harvey M., Slagle B. L. (1992). Mice deficient for p53 are developmentally normal but susceptible to spontaneous tumours. *Nature*.

[110] Tian X., Dai S., Sun J., Jiang S., Jiang Y. (2017). The association between the TP53 Arg72Pro polymorphism and colorectal cancer: an updated meta-analysis based on 32 studies. *Oncotarget*.

[111] Arber N., Hibshoosh H., Moss S. F. (1996). Increased expression of cyclin D1 is an early event in multistage colorectal carcinogenesis. *Gastroenterology*.

[112] Li Y., Wei J., Xu C., Zhao Z., You T. (2014). Prognostic significance of Cyclin D1 expression in colorectal cancer: a meta-analysis of observational studies. *PLoS One*.

[113] McKay J. A., Douglas J. J., Ross V. G., Curran S., Murray G. I., Cassidy J. (2000). Cyclin D1 protein expression and gene polymorphism in colorectal cancer. *International Journal of Cancer*.

